# The Role of MIF-173G/C Gene Polymorphism in the Susceptibility of Autoimmune Diseases

**DOI:** 10.1155/2020/7825072

**Published:** 2020-04-28

**Authors:** Xiangrong Du, Ruixia Li, Shoujun Song, Lei Ma, Haibo Xue

**Affiliations:** ^1^Department of Endocrinology and Metabolism, Binzhou Medical University Hospital, 661 Second Huanghe Road, Binzhou 256603, China; ^2^Department of Internal Medicine, Linzi District People's Hospital, No. 139 Huangong Road, Zibo 255400, China; ^3^Department of Endocrinology, Yantai Affiliated Hospital of Binzhou Medical University, No. 717 Jinfu Street, Yantai 264100, China; ^4^Department of Dermatology, Binzhou Medical University Hospital, No. 661 Second Huanghe Road, Binzhou 256603, China

## Abstract

Some certain genetic polymorphisms have been considered to implicate in the pathogenesis and progression of autoimmune diseases and may predispose to an early stage of general autoimmune susceptibility. Recent studies have been conducted to investigate the association between macrophage migration inhibitory factor- (MIF-) 173G/C gene polymorphism and autoimmune diseases; however, the results were not exactly identical. In the present study, a systematic review and meta-analysis of case-control studies was performed to estimate the relationship. A comprehensive search of PubMed, Ebsco, EMbase, WanFang databases and CNKI was done. Odds ratio (ORs) and corresponding 95% confidence intervals (CIs) were combined to pool the effect size. The publication bias was examined by Begg's funnel plots and Egger's test. RevMan 5.3 and STATA 12.0 software were used for statistical processing. 23 papers were included, and the results revealed that MIF-173G/C was significantly associated with an increased risk of autoimmune diseases in five genetic models (recessive genetic model: OR = 1.95, 95% CI: 1.52-2.50; dominant genetic model: OR = 1.35, 95% CI: 1.24-1.46; allele model: OR = 1.32, 95% CI: 1.23-1.41; homozygote model: OR = 1.92, 95% CI: 1.57-2.35; heterozygote model: OR = 4.92, 95% CI: 4.03-6.02), whether in Asia, Europe, or North America. Furthermore, subgroup analysis showed an increasing risk in rheumatoid arthritis (RA), ulcerative colitis (UC), Crohn's disease (CD), atopic dermatitis (AD), Henoch-Schonlein purpura (HSP), and Henoch-Schonlein purpura nephritis (HSPN), but it was not related to the susceptibility of autoimmune hepatitis (AIH). Therefore, it could be considered that MIF-173G/C polymorphism could increase the susceptibility of autoimmune diseases, while there may be the discrepancy of disease entity.

## 1. Introduction

Macrophage migration inhibitory factor (MIF) is a proinflammatory cytokine mainly released from Th2 cells and macrophages, which can mediate the host response to infection and stress by activating innate and adaptive immune pathways [1, 2]. In humans, MIF is encoded by a single gene located on chromosome 22q11.2m, a 12 kD peptide comprising 114 amino acids [3]. It mainly interacts with its receptor CD74 to form a complex with CD44, which can result in the lasted activation of the ERK-MAPK pathway by a Src tyrosine kinase signal transduction. Downstream effects of this pathway include NF*κ*B translocation to the nucleus, upregulation of PLA2 and prostaglandins, and stimulation of the arachidonic acid pathway [4]. So far, a single-nucleotide polymorphism in the 50 regions of the MIF gene, MIF-173G/C, a G to C transversion within the MIF promoter region at position -173 creates an AP4 transcription factor binding site [5, 6]. Previous studies have shown that MIF-173G/C polymorphism was related to the susceptibility of cancer [7], tuberculosis [8], and coronary atherosclerosis [9]. Recently, many studies have indicated that MIF-173G/C is associated with the pathogenesis and progression of autoimmune diseases.

The autoimmune diseases (AID) are characterized by dysfunction of the immune system leading to the loss of immune tolerance against self-tissues, the presence of autoreactive T and B cells, and a complex pathogenesis of multifactorial etiology, whereas genetic, epigenetic, and environmental factors are together responsible for the onset of AID [10, 11]. So far, we have come to understand that certain genetic factors, including genetic polymorphisms implicated in autoimmune diseases, may predispose to an early stage of general autoimmune susceptibility [12]. It has been a new hot spot about the relationship between MIF-173G/C and autoimmune diseases. In Graves' disease (GD), MIF-173G/C plays a dual effect, which is not only a risk factor for the morbidity of goiter but also a protective role in the development of untreated severe goiter [13]. It was considered that MIF-173G/C might play an important role in the susceptibility of UC, but not in CD [14]. Due to the divergence of the researches, we resolved on devising a meta-analysis to evaluate the relationship between them.

## 2. Materials and Methods

### 2.1. Literature Research

A comprehensive document search was conducted in the PubMed, Ebsco, Embase, and Chinese WanFang databases and China National Knowledge Infrastructure (CNKI) in view of the relationship between MIF-173G/C and autoimmune diseases from the inception to October 10, 2019. The search strategy used in the present study was “macrophage migration inhibitory factor OR MIF” AND “polymorphism OR variant OR mutation” AND “rs755266” AND “Autoimmune disease OR autoimmune disorder OR AD OR AID.” The language is restricted to English and Chinese, and the study manually retrieves references from the study and the latest review.

### 2.2. Eligibility Criteria

In the present meta-analysis, the included literatures must meet the flowing criteria: (1) case-control study or cohort study was published publicly; (2) the study must assess the association of MIF-173G/C gene polymorphism with the susceptibility of autoimmune diseases; (3) the experimental and control subjects involved in studies are human being; and (4) the odds ratios (ORs) and 95% confidence intervals (95% CIs) could be calculated by sufficient genotypic frequencies available in the studies. We applied the Preferred Reporting Items for Systematic Review and Meta-analysis statement (PRIMSA) for the meta-analysis.

### 2.3. Exclusion Criteria

We excluded the relevant documents according to the following standards: (1) duplicate publications, reviews, meta-analysis, letters, and editorial comments; (2) non-case-control study; (3) animal experiment; and (4) failure to offer the genotypic or allelic frequencies.

### 2.4. Data Extraction

Two investigators selected literatures independently by checking the title, abstract, and full text based on the eligibility and excluded criteria and ironed out the differences by discussing. And then the following contents were extracted: first author's name, year of publication, country of origin, disease species, genotyping method, the total number of controls and cases, the genotypic and allelic frequencies in controls and cases, and Hardy-Weinberg equilibrium (HWE) test results in controls.

The quality evaluation of literature involved in the present meta-analysis also was done by two investigators in the light of Newcastle-Ottawa Scale (NOS) [15]. We evaluated the quality from the following three aspects: subject selection, comparability of subjects, and clinical outcomes. The aggregate score is 9 points, and the research of high quality is more than 6 points.

### 2.5. Statistical Analysis

In the present study, HWE was used to evaluate the controls of all the included literatures by the chi-square test, and *P* > 0.05 was considered the genetic balance in the population. The pooled odds ratio (ORs) and corresponding 95% confidence intervals (CIs) were calculated to estimate the strength of the association between MIF-173G/C and autoimmune diseases in the following five genetic models: recessive genetic model (CC vs. GC+GG), dominant genetic model (CC+GC vs. GG), allelic genetic model (C vs. G), heterozygous genetic model (GC vs. GG), and homozygous genetic model (CC vs. GG). The conspicuous level of statistic was assessed by a *Z*-test with *P* < 0.05. The heterogeneity was assessed by Cochran's *Q* statistic and *I*-squared (*I*^2^) metric. The random effects model was selected when there is statistical heterogeneity with *P* < 0.10 or *I*^2^ > 50%. Otherwise, the fixed effects model is used for the merger analysis. We sequentially eliminated the single document to estimate the sensitivity by screening the OR and heterogeneity. Otherwise, subgroup analyses were performed by disease and area (Asian, Europe, and North America). Potential publication bias was assessed by Begg's funnel plots and Egger's test, and *P* < 0.05 was considered when there was significantly statistical publication bias. The calculation of ORs, 95% CI, heterogeneity, and sensitivity analysis was conducted by using the software Review manager (RevMan, version 5.3, Cochrane Collaboration, Copenhagen, Denmark). The subgroup analysis, Begg's funnel plots, and Egger's test were performed by the STATA 12.0 software (STATA Corp LP, College Station, USA).

## 3. Results

### 3.1. Characteristics of Eligible Studies

**Figure 1:**
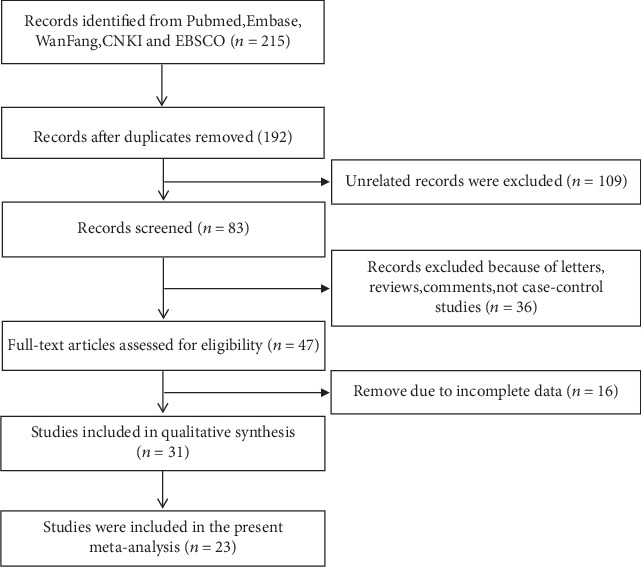
PRISMA flow diagram for the inclusion of studies in the meta-analysis.

In the initial search from PubMed, Ebsco, Embase, and Wanfang databases and CNKI, 215 potential records were identified based on our search strategy. All the retrieved research articles were screened manually by examining abstracts and texts; meanwhile, 192 publications were excluded in accordance to the exclusion criteria. Finally, 23 literatures [2, 14, 16–36] were included in the present meta-analysis.  Figure 1 shows the selection procedure: a total of twenty-nine human case-control trials were adopted, containing five databases of them which evaluated the relationship between MIF-173G/C and rheumatoid arthritis (RA) [16–20]. Three databases were Crohn's disease (CD) [14, 21, 22], ulcerative colitis (UC) [14, 22, 23], and autoimmune hepatitis (AIH) [2, 24], respectively. Two databases were about atopic dermatitis (AD) [25, 26], Henoch-Schonlein purpura nephritis (HSPN) [27], and Henoch-Schonlein purpura (HSP) [28]. The other nine diseases were inquired in single databases, including ankylosing spondylitis (AS) [29], primary gout [30], primary biliary cirrhosis (PBC) [24], psoriasis (Ps) [31], systemic lupus erythematosus (SLE) [32], Vogt-Koyanagi-Harada syndrome (VKH) [33], adult still disease (AOSD) [34], Behcet's disease (BD) [35], and scleroderma (SD) [36]. The areas of the studies involved in the present analysis were Asian, Europe, and North America. The genotyping method included the Polymerase Chain Reaction-Restriction Fragment Length Polymorphism (PCR-RFLP), PCR, and ELISA. All people frequencies of the controls were in HWE (*P* > 0.05). The quality scores of the involved studies were evaluated according to NOS, and all scores were more than 6 points. The analysis was conducted only when more than two articles were included in each subgroup. The characteristics of the involved studies are revealed in Table 1.

### 3.2. Results of Meta-analysis

**Figure 2:**
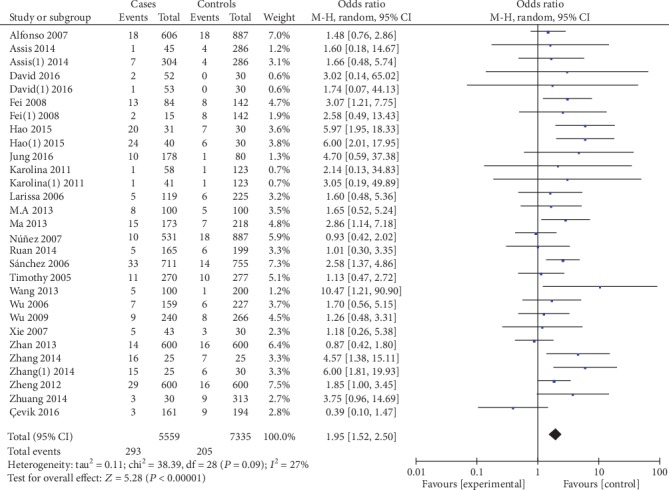
Association between MIF-173G/C gene polymorphism and autoimmune diseases in recessive model (CC/GC+GG).

**Figure 3:**
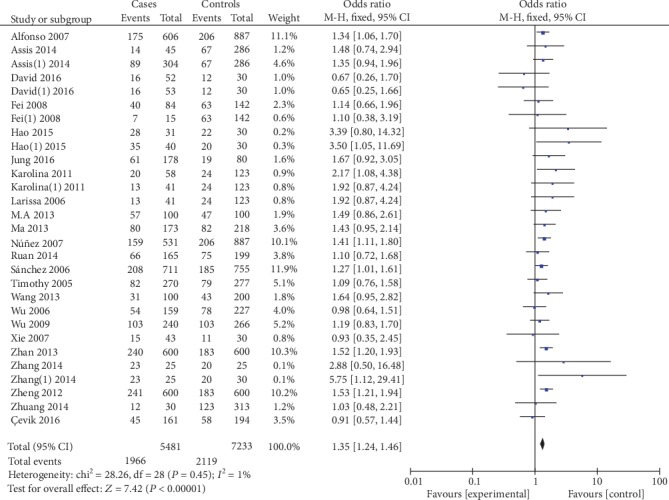
Association between MIF-173G/C gene polymorphism and autoimmune diseases in dominant model (CC+GC vs. GG).

**Figure 4:**
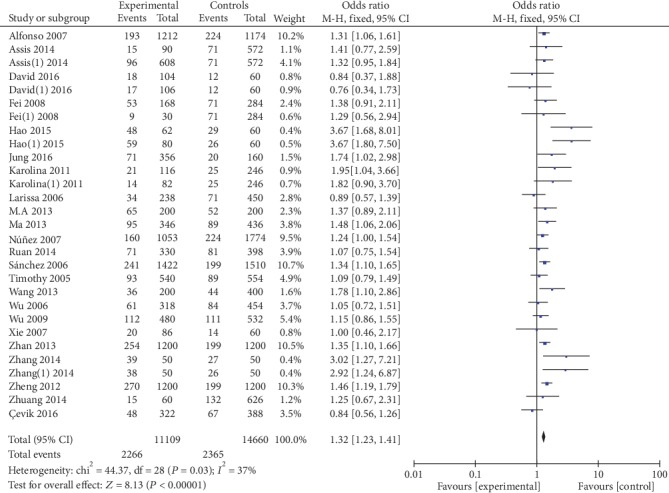
Association between MIF-173G/C gene polymorphism and autoimmune diseases in allelic model (C/G).

**Figure 5:**
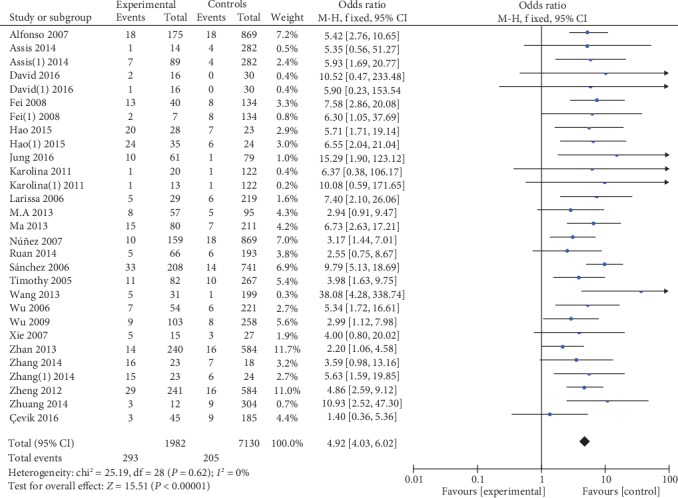
Association between MIF-173G/C gene polymorphism and autoimmune diseases in heterozygous model (GC/GG).

**Figure 6:**
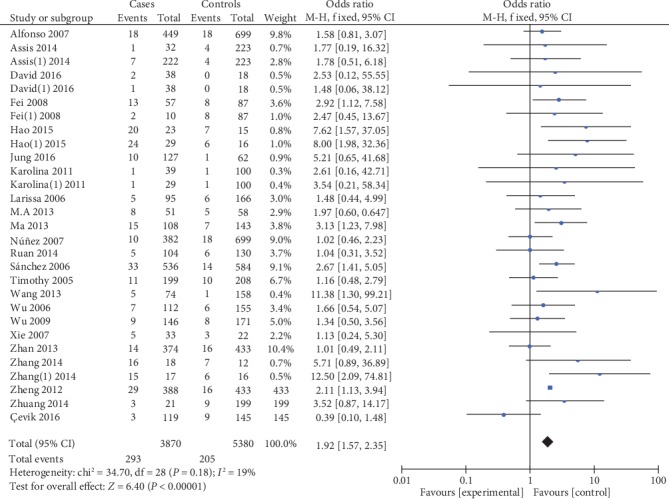
Association between MIF-173G/C gene polymorphism and autoimmune diseases in homozygous model (CC/GG).

5559 cases and 7335 controls were involved in the ultima meta-analysis. There was no significant heterogeneity between the MIF-173G/C polymorphism and autoimmune diseases (both *P* > 0.1), so the fixed effects model was used for meta-analysis. The polled summary crude odds radios (ORs) and corresponding 95% confidence intervals (CIs) in all genetic models were as follows: recessive genetic model (CC vs. GC+GG): OR = 1.95, 95%CIs = 1.52-2.50, *P* < 0.05; dominant genetic model (CC+GC vs. GG): OR = 1.35, 95%CIs = 1.24-1.46, *P* < 0.05; allelic genetic model (C vs. G): OR = 1.32, 95%CIs = 1.23-1.41, *P* < 0.05; heterozygous genetic model (GC vs. GG): OR = 4.92, 95%CIs = 4.03-6.02, *P* < 0.05; and homozygous genetic model (CC vs. GG): OR = 1.92, 95%CIs = 1.57-2.35, *P* < 0.05. The results are shown in Table 2 and Figures 2–6.

In addition, the results of the subgroup analysis performed according to area revealed the following data: in Asia, recessive genetic model ((CC vs. GC+GG): OR = 2.32, 95% CIs = 1.79-3.00, *P* < 0.05), dominant genetic model ((CC+GC vs. GG): OR = 1.38, 95% CIs = 1.23-1.53, *P* < 0.05), allelic genetic model ((C vs. G): OR = 1.40, 95% CIs = 1.28-1.54, *P* < 0.05), heterozygous genetic model ((GC vs. GG): OR = 1.29, 95% CIs = 1.15-1.44, *P* < 0.05), and homozygous genetic model ((CC vs. GG): OR = 1.91, 95% CIs = 1.55-2.38, *P* < 0.05); in Europe, recessive genetic model ((CC vs. GC+GG): OR = 1.58, 95% CIs = 1.09-2.29, *P* < 0.05), dominant genetic model ((CC+GC vs. GG): OR = 1.20, 95% CIs = 1.03-1.39, *P* < 0.05), allelic genetic model ((C vs. G): OR = 1.21, 95% CIs = 1.06-1.37, *P* < 0.05), heterozygous genetic model ((GC vs. GG): OR = 1.51, 95% CIs = 0.99-1.35, *P* = 0.70), and homozygous genetic model ((CC vs. GG): OR = 1.56, 95% CIs = 1.10-2.21, *P* < 0.05); in North America, recessive genetic model ((CC vs. GC+GG): OR = 1.21, 95% CIs = 0.73-2.00, *P* = 0.45), dominant genetic model ((CC+GC vs. GG): OR = 1.38, 95% CIs = 1.16-1.63, *P* < 0.05), allelic genetic model ((C vs. G): OR = 1.28, 95% CIs = 1.10-1.48, *P* < 0.05), heterozygous genetic model ((GC vs. GG): OR = 1.38, 95% CIs = 1.16-1.65, *P* < 0.05), and homozygous genetic model ((CC vs. GG): OR = 1.30, 95% CIs = 0.80-2.12, *P* = 0.29). The results are shown in Table 3.

In the analysis stratified by diseases, the results demonstrated the following: in AD, recessive genetic model (CC vs. GC+GG): OR = 3.03, 95% CIs = 1.34-6.83, *P* < 0.05; dominant genetic model (CC+GC vs. GG): OR = 1.29, 95% CIs = 1.04-1.59, *P* < 0.05; allelic genetic model (C vs. G): OR = 1.41, 95% CIs = 1.13-1.76, *P* < 0.05; homozygous genetic model (CC vs. GG): OR = 3.21, 95% CIs = 1.44-7.18, *P* < 0.05; and heterozygous genetic model (GC vs. GG) had no statistical significance. In RA, dominant genetic model (CC+GC vs. GG): OR = 1.20, 95% CIs = 1.01-1.43, *P* < 0.05; allelic genetic model (C vs. G): OR = 1.16, 95% CIs = 1.03-1.31, *P* < 0.05; recessive genetic model (CC vs. GC+GG); heterozygous genetic model (GC vs. GG); and homozygous genetic model (CC vs. GG) had no statistical significance. In UC, recessive genetic model (CC vs. GC+GG): OR = 1.93, 95% CIs = 1.01-3.01, *P* < 0.05; allelic genetic model (C vs. G): OR = 1.22, 95% CIs = 1.00-1.47, *P* < 0.05; dominant genetic model (CC+GC vs. GG); heterozygous genetic model (GC vs. GG); and homozygous genetic model (CC vs. GG) had no statistical significance. In CD, dominant genetic model (CC+GC vs. GG): OR = 1.30, 95% CIs = 1.10-1.52, *P* < 0.05; heterozygous genetic model (GC vs. GG): OR = 1.32, 95% CIs = 1.11-1.57, *P* < 0.05; allelic genetic model (C vs. G): OR = 1.23, 95% CIs = 1.04-1.47, *P* < 0.05; homozygous genetic model (CC vs. GG); and recessive genetic model (CC vs. GC+GG) had no statistical significance. In HSP, recessive genetic model (CC vs. GC+GG): OR = 2.60, 95% CIs = 1.55-4.37, *P* < 0.05; dominant genetic model (CC+GC vs. GG): OR = 1.26, 95% CIs = 1.05-1.51, *P* < 0.05; allelic genetic model (C vs. G): OR = 1.59, 95% CIs = 1.28-1.98, *P* < 0.05; homozygous genetic model (CC vs. GG): OR = 1.88, 95% CIs = 1.25-2.82, *P* < 0.05; and heterozygous genetic model (GC vs. GG) had no statistical significance. In HPSN, recessive genetic model (CC vs. GC+GG): OR = 2.88, 95% CIs = 1.72-4.83, *P* < 0.05; dominant genetic model (CC+GC vs. GG): OR = 1.27, 95% CIs = 1.06-1.53, *P* < 0.05; allelic genetic model (C vs. G): OR = 1.65, 95% CIs = 1.33-2.05, *P* < 0.05; homozygous genetic model (CC vs. GG): OR = 2.03, 95% CIs = 1.32-3.12, *P* < 0.05; and heterozygous genetic model (GC vs. GG) had no statistical significance. And there was no significant association between the AIH and MIF-173G/C gene polymorphisms. The results are shown in Table 4.

### 3.3. Sensitivity Analysis

After eliminating any research, the total effect of meta-analysis did not change significantly, indicating that the results were stable and credible.

### 3.4. Publication Bias

As shown in Tables 2–4, Egger's and Begg's tests were conducted to detect the publication bias in the present meta-analysis. Whether in the total analysis or in the subgroup of area and disease, the *P* values of Egger's and Begg's tests were all greater than 0.05, which demonstrated that the present study had no publication bias.

## 4. Discussion and Conclusion

MIF is an immunoregulatory cytokine secreted from various types of cells in different tissues, which can promote leukocyte recruitment and subsequently promote the expression and function of multiple cytokines and chemokines, including tumor necrosis factor (TNF), interleukin-6 (IL-6), CXCL1, and CCL2 [37]. Recently, MIF has been reported to be a key response regulator which can directly activate immune cells or participate in activation pathways initially triggered by other factors [38]. A functional single-nucleotide polymorphism (SNP) was identified in the untranslated 5′region of MIF gene at position -173 consisting of a G to C transition [39]. The new research about the variate action of MIF-173G/C in different diseases was conducted and revealed that MIF-173G/C polymorphism could weakly mediate the development of metabolic syndrome and significantly predict the risk of death by inducing low-grade inflammation independently in Turkish man, but not in woman [40]. Furthermore, the carriage of MIF-173C was associated with unfavorable outcome and death in pneumococcal meningitis, demonstrating strongly that MIF alleles were a genetic marker of morbidity and mortality of pneumococcal meningitis [41].

At present, more and more researchers pay more attention to the roles of MIF-173G/C in autoimmune diseases. The level of MIF-173G/C in progressive multiple sclerosis (MS) is significantly increased in male, suggesting that it can be a sex-specific disease modifier and its receptor CD74 signaling might provide an effective, trackable therapeutic approach for MS subjects of two sexes [42]. In systemic lupus erythematosus (SLE), MIF-173C genotype may be a protective factor. However, the high expression of MIF polymorphisms is associated with an increased incidence of end-organ injury in Caucasians and African Americans [43]. And another study reported that MIF-173CC allele might increase the risk for RA, especially among CRP-negative patients in China [44]. In order to systematically elucidate the relationship between MIF-173G/C and autoimmune diseases, we designed the present meta-analysis.

All the 23 articles were brought into the present meta-analysis, including 18 papers written in English and 5 papers written in Chinese [20, 23, 27, 28, 30]. Two of the Chinese literatures [20, 23] are dissertations with high quality. The total results revealed that a strong correlation with pathogenesis of autoimmune disease, whether in the recessive genetic model (OR = 1.95, 95% CI = 1.52 to 2.50), dominant genetic model (OR = 1.35, 95% CI = 1.24 to 1.46), allelic genetic model (OR = 1.32, 95% CI = 1.23 to 1.41), heterozygous genetic model (OR = 4.92, 95% CI = 4.03 to 6.02), or homozygous genetic model (OR = 1.92, 95% CI = 1.57 to 2.35). When the MIF-173 GG genotypes were used as the reference group, the GC heterozygous genotype was associated with a significant 4.92-fold increasing susceptibility to autoimmune diseases, particularly in the heterozygous model. Therefore, these data indicated that MIF-173G/C could significantly increase the susceptibility of autoimmune diseases, especially the MIF-173GC genotype.

The subgroups categorized by area demonstrated significant associations between MIF-173G/C and autoimmune diseases in Asia, Europe, and North America. In Asia, a strong pooled OR was detected in the recessive model (OR = 2.32, 95% CI = 1.79 to 3.00), dominant model (OR = 1.38, 95% CI = 1.23 to 1.53), allelic model (OR = 1.40, 95% CI = 1.28 to 1.54), heterozygous model (OR = 1.29, 95% CI = 1.15 to 1.44), or homozygous model (OR = 1.91, 95% CI = 1.55 to 2.38). Nevertheless, only 4 genetic models including the recessive model (OR = 1.20, 95% CI = 1.03 to 1.39), dominant model (OR = 1.58, 95% CI = 1.09 to 2.29), allelic model (OR = 1.21, 95% CI = 1.06 to 1.37), and homozygous model (OR = 1.56, 95% CI = 1.10 to 2.21) had statistical significance in Europe. In North America, the dominant model (OR = 1.38, 95% CI = 1.16 to 1.63), allelic genetic model (OR = 1.28, 95% CI = 1.10 to 1.48), heterozygous genetic model (OR = 1.38, 95% CI = 1.16 to 1.65), and homozygous genetic model (OR = 1.92, 95% CI = 1.57 to 2.35) showed statistical significance. The abovementioned results indicated that the high expression of MIF-173G/C could increase the prevalence of autoimmune diseases whether in Asia, Europe, or North America. Additionally, the association was detected in all 5 genetic models especially in Asia, which revealed that MIF-173G/C could increase the susceptibility of autoimmune diseases more significantly compared with that in Europe and North America. Therefore, it can be considered that there may be regional differences.

Moreover, it was found that MIF-173G/C was associated with the increased risk of RA in two genetic models including the recessive model (OR = 1.20, 95% CI = 1.01 to 1.43) and allelic model (OR = 1.16, 95% CI = 1.03 to 1.31). In UC, the allelic model (OR = 1.22, 95% CI = 1.00 to 1.47) and dominant model (OR = 1.93, 95% CI = 1.01 to 3.01) showed significant association, respectively. Three genetic models including the dominant model (OR = 1.30, 95% CI = 1.10 to 1.52), allelic model (OR = 1.23, 95% CI = 1.04 to 1.47), and heterozygous model (OR = 1.32, 95% CI = 1.11 to 1.57) had been demonstrated statistical significances in CD. In the subgroup of AD, four genetic models including the recessive model (OR = 3.03, 95% CI = 1.34 to 6.83), dominant model (OR = 1.41, 95% CI = 1.13 to 1.76), allelic model (OR = 1.29, 95% CI = 1.04 to 1.59), and homozygous model (OR = 3.21, 95% CI = 1.44 to 7.18) had been confirmed to be significantly different. Similar results of HSP were found in the recessive model (OR = 2.60, 95% CI = 1.55 to 4.37), dominant model (OR = 1.26, 95% CI = 1.05 to 1.51), allelic model (OR = 1.59, 95% CI = 1.28 to 1.98), and homozygous model (OR = 1.88, 95% CI = 1.25 to 2.82). The HSPN subgroup revealed equal numbers of genetic models to HSP, including the recessive model (OR = 2.88, 95% CI = 1.72 to 4.83), dominant model (OR = 1.65, 95% CI = 1.33 to 2.05), allelic model (OR = 1.27, 95% CI = 1.06 to 1.53), and homozygous model (OR = 2.03, 95% CI = 1.32 to 3.12). In AIH patients, there was no significant association in any genetic model. Therefore, it can be indicated that MIF-173G/C may play different roles in the pathogenesis of different autoimmune diseases, based on the stabilities in sensitivity analyses and no publication bias in all included studies.

In conclusion, the present study verified that there was a significant relationship between MIF-173G/C single-nucleotide polymorphism and the susceptibility of autoimmune diseases, whether in Asia, Europe, or North America. MIF-173G/C can be used as a potential therapeutic target in the treatment prescription of autoimmune diseases. However, in different autoimmune diseases, MIF-173G/C fulfilled various functions. So well-designed studies with larger sample size are needed to explore the specific mechanism in which MIF-173G/C affects the pathogenesis of different autoimmune diseases.

## Figures and Tables

**Table 1:** Characteristics of the included studies.

First author	Year	Disease	Country	Area	Method	Case			Control			*P*_HWE_
						GG	GC	CC	GG	GC	CC	
Alfonso	2007	RA	Mexico	North America	PCR-RFLP	431	157	18	681	188	18	0.24
Assis (1)	2014	AIH	America	Europe	ELISA	31	13	1	219	63	4	0.83
Assis (2)	2014	PBC	America	Europe	ELISA	215	82	7	219	63	4	0.83
Fei (1)	2008	UC	China	Asia	PCR-RFLP	44	27	13	79	55	8	0.7
Fei (2)	2008	CD	China	Asia	PCR-RFLP	8	5	2	79	55	8	0.7
Nunez	2007	CD	Spain	Europe	NA	372	149	10	681	188	18	0.24
Çevik	2016	AS	Turkey	Europe	RFLP	116	42	3	136	49	9	0.11
Zhan	2013	VKH	China	Asia	PCR-RFLP	360	226	14	417	167	16	0.88
David (1)	2016	AIH	Japan	Asia	ELISA	36	14	2	18	12	0	0.17
David (2)	2016	AIH	America	Europe	ELISA	37	15	1	18	12	0	0.17
Sanchez	2006	SLE	Spain	Europe	PCR-RFLP	503	175	33	570	171	14	0.78
Wang	2013	AOSD	China	Asia	PCR-RFLP	69	26	5	157	42	1	0.31
Wu	2009	Ps	China	Asia	PCR-RFLP	137	94	9	163	95	8	0.8
Jung	2016	AD	Korea	Asian	PCR-RFLP	117	51	10	61	18	1	0.18
Karolina (1)	2011	UC	Poland	Europe	PCR-RFLP	38	19	1	99	23	1	0.79
Karolina (2)	2011	CD	Poland	Europe	PCR-RFLP	28	12	1	99	23	1	0.79
Larissa	2006	RA	Germany	Europe	PCR	90	24	5	160	59	6	0.84
Ma	2013	AD	China	Asia	NA	93	65	15	136	75	7	0.39
M.A.	2013	RA	Mexico	North America	PCR-RFLP	43	49	8	53	42	5	0.36
Wu	2006	SD	America	Europe	ELISA	105	47	7	149	72	6	0.44
Timothy	2005	RA	Holland	Europe	PCR	188	71	11	198	69	10	0.20
Zheng	2012	BD	China	Asia	PCR-RFLP	359	212	29	417	167	16	0.88
Hao (1)	2015	HSPN	China	Asia	PCR-RFLP	3	8	20	8	15	7	1.00
Hao (2)	2015	HSPN	China	Asia	PCR-RFLP	5	11	24	10	14	6	0.79
Ruan	2014	UC	China	Asia	NA	99	61	5	124	69	6	0.33
Xie	2007	RA	China	Asia	PCR	28	10	5	19	8	3	0.16
Zhang (1)	2014	HSP	China	Asia	PCR-RFLP	2	7	16	5	13	7	0.82
Zhang (2)	2014	HSP	China	Asia	PCR-RFLP	2	8	15	10	14	6	0.79
Zhuang	2014	PG	China	Asia	PCR-RFLP	18	9	3	190	114	9	0.09

RA: rheumatoid arthritis; AIH: autoimmune hepatitis; PBC: primary biliary cirrhosis; UC: ulcerative colitis; CD: Crohn's disease; AS: ankylosing spondylitis; VKH: Vogt-Koyanagi-Harada syndrome; SLE: systemic lupus erythematosus; AOSD: adult Still disease; Ps: psoriasis; AD: atopic dermatitis; SD: scleroderma; BD: Behcet's disease; HSPN: Henoch-Schonlein purpura nephritis; HSP: Henoch-Schonlein purpura; PG: primary gout; PCR-RFLP: Polymerase Chain Reaction-Restriction Fragment Length Polymorphism; PCR: Polymerase Chain Reaction; NA: not available.

**Table 2:** The summary of the results from different comparative genetic models in all subjects.

Genetic models	*I* ^2^ (%)	*P*_I_	Effects model	OR (95% CI)	*Z*	*P*_z_	Egger's regression analysis	*P*_E_	Begg's regression analysis	*P*_B_
C/G	37	0.03	FIX	1.32 (1.23, 1.41)	8.13	0.01	0.14	0.89	0.36	0.72
CC/GG	19	0.18	FIX	1.92 (1.57, 2.35)	6.40	0.01	0.80	0.43	1.41	0.16
GC/GG	0	0.62	FIX	4.92 (4.03, 6.02)	15.51	0.01	-1.89	0.07	1.07	0.29
CC+GC/GG	1	0.45	FIX	1.35 (1.24, 1.46)	7.42	0.01	0.21	0.83	0.92	0.36
CC/GC+GG	27	0.09	FIX	1.95 (1.52, 2.50)	5.28	0.01	1.04	0.31	0.73	0.46

**Table 3:** The summary of the results from different comparative genetic models in different areas.

Areas	Genetic models	*I* ^2^ (%)	*P*_I_	Effects model	OR (95% CI)	*Z*	*P*_z_	Egger's regression analysis	*P*_E_	Begg's regression analysis	*P*_B_
Asia	C/G	49.5	0.01	FIX	1.40 (1.28, 1.54)	7.28	0.01	-0.22	0.79	-0.87	0.38
	CC/GG	0	0.84	FIX	1.91 (1.55, 2.38)	6.03	0.01	1.79	0.15	1.10	0.27
	GC/GG	0	0.57	FIX	1.29 (1.15, 1.44)	4.39	0.01	-1.10	0.03	-1.78	0.08
	CC+GC/GG	14.1	0.29	FIX	1.38 (1.23, 1.53)	5.76	0.01	0.34	0.39	0.42	0.68
	CC/GC+GG	26.3	0.15	FIX	2.32 (1.79, 3.00)	6.42	0.01	1.46	0.29	0.87	0.38

Europe	C/G	27.8	0.20	FIX	1.21 (1.06, 1.37)	2.87	0.01	1.95	0.30	0.42	0.68
	CC/GG	5.3	0.39	FIX	1.56 (1.10, 2.21)	2.52	0.01	0.16	0.92	0	1
	GC/GG	11.3	0.34	FIX	1.51 (0.99, 1.35)	1.81	0.70	1.52	0.36	0	1
	CC+GC/GG	14.9	0.31	FIX	1.20 (1.03, 1.39)	2.36	0.01	-0.02	0.81	1.04	0.30
	CC/GC+GG	10	0.35	FIX	1.58 (1.09, 2.29)	2.43	0.02	-0.22	0.89	-0.21	0.84

North America	C/G	0	0.72	FIX	1.28 (1.10, 1.48)	3.17	0.01	-8.79	___	-1.00	0.32
	CC/GG	0	0.40	FIX	1.30 (0.80, 2.12)	1.05	0.29	-8.79	___	-1.00	0.32
	GC/GG	0	0.59	FIX	1.38 (1.16, 1.65)	3.64	0.01	134.28	___	1.00	0.32
	CC+GC/GG	0	0.77	FIX	1.38 (1.16, 1.63)	3.71	0.01	0.08	___	1.00	0.32
	CC/GC+GG	0	0.37	FIX	1.21 (0.73, 2.00)	0.75	0.45	-0.87	___	-1.00	0.32

**Table 4:** The summary of the results from different comparative genetic models in different diseases.

Diseases	Genetic models	*I* ^2^ (%)	*P*_I_	Effects model	OR (95% CI)	*Z*	*P*_z_	Egger's regression analysis	*P*_E_	Begg's regression analysis	*P*_B_
RA	C/G	0	0.51	FIX	1.16 (1.03, 1.31)	2.37	0.02	-1.76	0.18	-0.98	0.33
	CC/GG	0	0.96	FIX	1.43 (0.95, 2.14)	1.72	0.09	-0.40	0.75	-0.49	0.62
	GC/GG	13.5	0.33	FIX	1.12 (0.99, 1.28)	1.76	0.08	-1.87	0.14	-1.47	0.14
	CC+GC/GG	11.9	0.34	FIX	1.20 (1.01, 1.43)	2.12	0.03	-2.03	0.13	-0.98	0.33
	CC/GC+GG	0	0.98	FIX	1.38 (0.92, 2.07)	1.53	0.13	-0.06	0.95	0	1.00

UC	C/G	32	0.23	FIX	1.22 (1.00, 1.47)	1.99	0.05	5.19	0.21	1.57	0.12
	CC/GG	0	0.47	FIX	1.86 (0.98, 3.50)	1.91	0.06	-0.49	0.89	-0.52	0.60
	GC/GG	51.6	0.13	FIX	1.11 (0.91, 1.38)	1.50	0.14	4.85	0.44	0.52	0.60
	CC+GC/GG	29.3	0,24	FIX	1.15 (0.96, 1.38)	1.50	0.14	5.74	0.10	1.57	0.12
	CC/GC+GG	0	0.39	FIX	1.93 (1.01, 3.01)	1.99	0.05	-1.23	0.77	-0.52	0.60

CD	C/G	0	0.58	FIX	1.23 (1.04, 1.47)	2.40	0.02	1.14	0.53	1.57	0.12
	CC/GG	0	0.49	FIX	1.22 (0.64, 2.33)	0.60	0.55	2.66	0.13	1.57	0.12
	GC/GG	0	0.54	FIX	1.32 (1.11, 1.57)	3.14	0.01	-0.19	0.92	-0.52	0.60
	CC+GC/GG	0	0.68	FIX	1.30 (1.10, 1.52)	3.09	0.01	0.24	0.90	0.52	0.60
	CC/GC+GG	0	0.42	FIX	1.14 (0.59, 2.18)	0.38	0.7	2.53	0.24	0.52	0.60

AIH	C/G	0	0.40	FIX	1.03 (0.73, 1.45)	0.17	0.86	-6.57	0.03	-1.57	0.12
	CC/GG	0	0.97	FIX	1.86 (0.40, 8.75)	0.79	0.43	0.31	0.87	-0.52	0.60
	GC/GG	39.8	0.19	FIX	0.93 (0.67, 1.29)	0.43	0.67	-10.62	0.04	-1.57	0.12
	CC+GC/GG	27.4	0.25	FIX	0.98 (0.71, 1.33)	0.16	0.87	-9.52	0.01	-1.57	0.12
	CC/GC+GG	0	0.95	FIX	1.98 (0.42, 9.32)	0.87	0.39	1.39	0.61	0.52	0.60

AD	C/G	0	0.52	FIX	1.41 (1.13, 1.76)	3.03	0.01	2.37	____	1.00	0.32
	CC/GG	0	0.62	FIX	3.21 (1.44, 7.18)	2.84	0.01	3.42	____	1.00	0.32
	GC/GG	0	0.60	FIX	1.21 (0.96, 1.52)	1.6	0.11	1.67	____	1.00	0.32
	CC+GC/GG	0	0.67	FIX	1.29 (1.04, 1.59)	2.38	0.02	2.02	____	1.00	0.32
	CC/GC+GG	0	0.65	FIX	3.03 (1.34, 6.83)	2.67	0.01	3.02	____	1.00	0.32

AP	C/G	0	0.39	FIX	1.59 (1.28, 1.98)	.12	0.01	18.13	____	1.00	0.32
	CC/GG	9.7	0.29	FIX	1.88 (1.25, 2.82)	3.03	0.01	10.79	____	1.00	0.32
	GC/GG	0	0.46	FIX	1.22 (0.89, 1.68)	1.21	0.23	82.99	____	1.00	0.32
	CC+GC/GG	0	0.57	FIX	1.26 (1.05, 1.51)	2.40	0.02	8.90	____	1.00	0.32
	CC/GC+GG	0	0.61	FIX	2.60 (1.55, 4.37)	3.60	0.01	15.55	____	1.00	0.32

HSPN	C/G	0	0.78	FIX	1.65 (1.33, 2.05)	4.54	0.01	8.39	____	1.00	0.32
	CC/GG	0	0.70	FIX	2.03 (1.32, 3.12)	3.21	0.01	7.45	____	1.00	0.32
	GC/GG	0	0.87	FIX	1.15 (0.82, 1.61)	0.81	0.42	34.18	____	1.00	0.32
	CC+GC/GG	0	0.97	FIX	1.27 (1.06, 1.53)	2.63	0.01	4.63	____	1.00	0.32
	CC/GC+GG	0	0.88	FIX	2.88 (1.72, 4.83)	4.02	0.01	7.69	____	1.00	0.32

## Data Availability

The data used to support the findings of this study are available from the corresponding author upon request.

## Abbreviations

MIF: Macrophage migration inhibitory factor
AID: Autoimmune diseases
ORs: Odds ratio
95% CIs: 95% of confidence intervals
RA: Rheumatoid arthritis
UC: Ulcerative colitis
CD: Crohn's disease
AD: Atopic dermatitis
HSP: Henoch-Schonlein purpura
HSPN: Henoch-Schonlein purpura nephritis
AIH: Autoimmune hepatitis
GD: Graves' disease
CNKI: China National Knowledge Infrastructure
PRIMSA: Preferred Reporting Items for Systematic Review and Meta-analysis statement
HWE: Hardy-Weinberg equilibrium
NOS: Newcastle-Ottawa Scale
PCR-RFLP: Polymerase Chain Reaction-Restriction Fragment Length Polymorphism
SLE: Systemic lupus erythematosus.
